# Identification of Plasma Glycosphingolipids as Potential Biomarkers for Prostate Cancer (PCa) Status

**DOI:** 10.3390/biom10101393

**Published:** 2020-09-30

**Authors:** Ashley J. Snider, Michael C. Seeds, Laurel Johnstone, Justin M. Snider, Brian Hallmark, Rahul Dutta, Cristina Moraga Franco, John S. Parks, Jeannette T. Bensen, Corey D. Broeckling, James L. Mohler, Gary J. Smith, Elizabeth T.H. Fontham, Hui-Kuan Lin, William Bresette, Susan Sergeant, Floyd H. Chilton

**Affiliations:** 1 Department of Nutritional Sciences, College of Agriculture and Life Sciences, University of Arizona, Tucson, AZ 85721, USA; ashleysnider@arizona.edu (A.J.S.); laureljo@arizona.edu (L.J.); justinsnider@arizona.edu (J.M.S.); bhallmark@statlab.bio5.org (B.H.); cristinam@email.arizona.edu (C.M.F.); bresette@email.arizona.edu (W.B.); 2 Stony Brook Cancer Center, Stony Brook University, Stony Brook, NY 11794, USA; 3 Wake Forest Institute of Regenerative Medicine, Wake Forest School of Medicine, Winston-Salem, NC 27101, USA; mseeds@wakehealth.edu; 4 Department of Internal Medicine-Molecular Medicine, Wake Forest School of Medicine, Winston-Salem, NC 27157, USA; jparks@wakehealth.edu; 5 Department of Urology, Wake Forest Baptist Health, Winston-Salem, NC 27103, USA; rdutta@wakehealth.edu; 6 North Carolina and Louisiana Prostate Cancer Program, Lineberger Comprehensive Cancer Center, University of North Carolina, Chapel Hill, NC 27599, USA; jeannette_bensen@med.unc.edu; 7 Proteomics and Metabolomics Facility, Colorado State University, Fort Collins, CO 80523, USA; Corey.Broeckling@ColoState.EDU; 8 Department of Urology, Roswell Park Comprehensive Cancer Center, Buffalo, NY 14263, USA; James.Mohler@roswellpark.org (J.L.M.); Gary.Smith@roswellpark.org (G.J.S.); 9 School of Public Health, Louisiana State University Health Sciences Center, New Orleans, LA 70112, USA; EFonth@lsuhsc.edu; 10 Cancer Biology Comprehensive Cancer Center, Wake Forest Baptist Medical Center, Winston-Salem, NC 27101, USA; hulin@wakehealth.edu; 11 Department of Biochemistry, Wake Forest School of Medicine, Winston-Salem, NC 27157, USA; ssergean@wakehealth.edu; 12 Department of Cancer Biology, Wake Forest School of Medicine, Winston-Salem, NC 27101, USA

**Keywords:** sphingolipid, ceramide, biomarker, lipidomic, metabolomic

## Abstract

Prostate cancer (PCa) is the most common male cancer and the second leading cause of cancer death in United States men. Controversy continues over the effectiveness of prostate-specific antigen (PSA) for distinguishing aggressive from indolent PCa. There is a critical need for more specific and sensitive biomarkers to detect and distinguish low- versus high-risk PCa cases. Discovery metabolomics were performed utilizing ultra-performance liquid chromatography-tandem mass spectrometry (UPLC-MS) on plasma samples from 159 men with treatment naïve prostate cancer participating in the North Carolina-Louisiana PCa Project to determine if there were metabolites associated with aggressive PCa. Thirty-five identifiable plasma small molecules were associated with PCa aggressiveness, 15 of which were sphingolipids; nine common molecules were present in both African-American and European-American men. The molecules most associated with PCa aggressiveness were glycosphingolipids; levels of trihexosylceramide and tetrahexosylceramide were most closely associated with high-aggressive PCa. The Cancer Genome Atlas was queried to determine gene alterations within glycosphingolipid metabolism that are associated with PCa and other cancers. Genes that encode enzymes associated with the metabolism of glycosphingolipids were altered in 12% of PCa and >30% of lung, uterine, and ovarian cancers. These data suggest that the identified plasma (glyco)sphingolipids should be further validated for their association with aggressive PCa, suggesting that specific sphingolipids may be included in a diagnostic signature for PCa.

## 1. Introduction

Prostate cancer (PCa) is the most diagnosed cancer in men, accounting for over 350,000 deaths worldwide in 2018 [1,2,3]. Prostate cancer is still subject to substantial overdiagnosis and therefore screening tests are needed that more accurately identify men with aggressive cancer [4]. Prostate-specific antigen (PSA) became the standard test for PCa early detection in the 1990s. However, recent studies comparing PSA screened men to those without screening revealed relatively small differences in PCa mortality [5,6,7]. The Gleason grading system is a tool for determining PCa aggressiveness [8], and is composed of a primary grade (histological features of the largest tumor area) and a secondary grade (the next largest area) and has a five-tiered Grade Group system that includes 1: Gleason ≤ 6; 2: Gleason 7 (3 + 4); 3: Gleason 7 (4 + 3); 4: Gleason 8; 5: Gleason 9 or 10. PCa that is Grade Group 1 are considered low-risk, indolent tumors while Grade Groups 4 and 5 predict poor prognosis and overall survival. Gleason Score 7 tumors have highly variable clinical outcomes, with Grade Group 3 associated with a three-fold increase in mortality compared to Grade Group 2 [8,9,10]. An estimated 80–85% of men between 70 and 80 years of age with PCa have Gleason Scores 7–10 [11]. The uncertainty of PSA as an early detection tool and the wide variability in outcomes for certain Grade Groups warrants the search for better biomarkers to determine PCa aggressiveness.

Discovery metabolomics and other unbiased approaches to molecular analysis are beginning to fill critical roles in defining precipitating events in cancer development, identifying molecular indicators of the cancer trajectory, and predicting targeted treatment strategies that prolong lifespan and improve overall quality of life. Two recent reviews examined the potential role of metabolites and molecular signatures in blood, urine, (tumor) tissues, or extracellular vesicles in PCa [12,13]. Studies of circulating plasma lipids in PCa have focused on unsaturated fatty acids, cholesterol, phospholipids, and lyso-phospholipids [12,14,15,16]. However, discovery metabolomics is a young field with significant technical, methodological, and analytical hurdles [13]. There is a clear need for more definitive studies that focus on metabolic networks (pathways) underpinning PCa occurrence and aggressiveness. 

**Figure 1:**
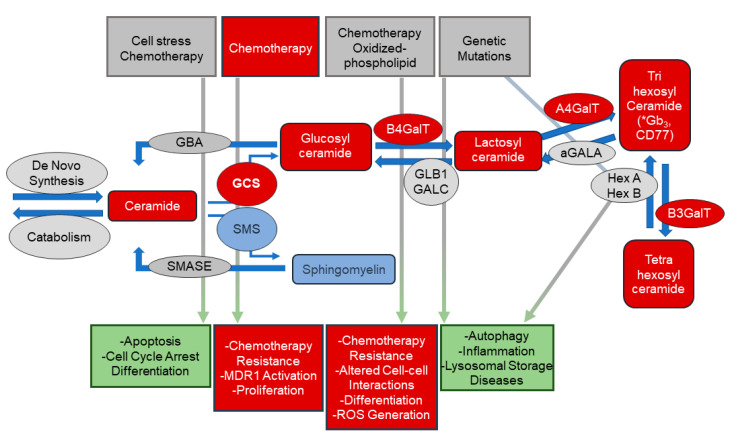
Overview of sphingolipid metabolism and biology. This scheme depicts stimuli and biologies associated with bioactive sphingolipids in the regulation of critical cellular biologic processes. Ceramides, and other sphingolipids, are important signaling molecules in numerous processes including apoptosis and cell cycle arrest. Upregulation of the pathways converting ceramide to complex glycosphingolipids (via the addition of sugar moieties) or to sphingomyelin (via the addition of phosphocholine) decreasing cellular ceramide levels may allow cancer cells to escape apoptosis. Increased levels of glycosphingolipids have been implicated in multi-drug resistance (MDR) in cancer cells. The metabolic pathways that generated these bioactive lipids are tightly regulated. Enzyme expression and/or activity are altered by several exogenous stimuli and the resulting alterations in lipid levels result in numerous cellular and biologic responses. Sphingolipids (and their associated metabolic enzymes) identified in plasma from PCa patients using untargeted metabolomic analyses are indicated in red. * putative IDs based on glycosylation patterns. αGALA: alpha-galactosidase; A4GalT: lactosylceramide 4-alpha-galactosyltransferase; B3GALT: beta-1,3-galctosyltransferase; B4GalT: beta-1,4-galctosyltransferase 1; GALC: galactosylcerbrosidase; GBA: glucosylceramidase; GCS: glucosylceramide synthase; HexA/HexB: hexosaminidase alpha/beta; SMASE: sphingomyelinase; SMS: sphingomyelin synthase.

Recent studies have suggested altered sphingolipid levels, specifically glycosphingolipids and their metabolic enzymes, may play critical roles in initiation and malignant transformation of numerous cancers [17]. Sphingolipids were understood to have a primarily structural role in cellular membranes, with critical roles in cancer signaling and biology now being recognized for individual sphingolipids, including glycosphingolipids [18,19] (Figure 1). Herein our data identify specific glycosphingolipid species to be highly associated with aggressive PCa, suggesting that further analysis of sphingolipids may help identify a novel diagnostic for PCa and play a role in determining PCa aggressiveness in African- and European-American men. 

## 2. Materials and Methods

### 2.1. Cohort

The North Carolina Louisiana Prostate Cancer Project (PCaP) is a longitudinal study that includes self-reported African-American and European-American participants with a diagnosis of PCa [20]. Retrospective, de-identified plasma samples were analyzed from 80 African-American (AfAm) and 79 European-American (EuAm) men obtained prior to treatment for PCa (Table 1) in accordance with a Wake Forest Health Sciences Investigational Review Board approved protocol. Written informed consent was obtained from all research subjects. Additional details about the study methods and design were published [20].

### 2.2. Main Comparisons of Interest: Plasma Metabolites and PCa Aggressiveness

The correlation between levels of plasma metabolites and PCa aggressiveness [20], which was classified based on clinical Grade Group and clinical stage from prostatectomy specimens, as well as PSA at diagnosis such that: (1) Aggressive = Grade Group 4 or 5, or PSA > 20 ng/ mL, or Grade Group 2 or 3, and stage cT3–cT4; (2) Low Aggressive = Grade Group 1 and stage cT1–cT2, and PSA < 10 ng/mL; (3) Intermediate Aggressive = all other cases. Comparisons with biomarkers were evaluated for the PCaP Aggressiveness score and Grade Groups 1 vs. 2–5. 

### 2.3. Sample Preparation 

A total of 50 µL of each plasma sample was extracted with 190 µL of LCMS grade methanol containing 10 µL of SPLASH LipidoMIX Internal standard mixture (Avanti, Al). Samples were vortexed (4 °C for 30 min), centrifuged (13,000 RPM at 4 °C for 15 min), and supernatant evaporated under nitrogen gas before resuspension in 100 µL of LCMS grade methanol. Quality Control (QC) mixtures were created for plasma samples by pooling 10 µL from each sample. 

### 2.4. UPLC-MS Analysis

In total, 90 µL of extract was dried under nitrogen and suspended in 100 μL of toluene/methanol (3/2, *v*/*v*). Then, 3 μL of extract was injected onto a Waters Acquity UPLC system (Milford, MA, USA) in randomized order, and separated using a Waters Acquity UPLC CSH Phenyl Hexyl column (1.7 µM, 1.0 × 100 mm), using a gradient from solvent A (water, 0.1% formic acid) to solvent B (Acetonitrile, 0.1% formic acid). Injections were made in 100% A, held at 100% A for 1 min, ramped to 98% B over 12 min, held at 98% B for 3 min, and re-equilibrated utilizing a 200 µL/min flow rate. The column and samples were held at 65 and 6 °C, respectively. Eluent was infused via electron spray ionization (ESI) source into a Waters Xevo G2 Q-TOF-MS (Milford, MA, USA) in positive mode, scanning 50–2000 *m*/*z* at 0.2 s per scan, and alternated between MS (6 V collision energy) and MSE mode (15–30 V ramp). Calibration was performed using sodium iodide with 1 ppm mass accuracy. Capillary voltage was 2200 V, source temperature was 150 °C, and nitrogen desolvation temperature was 350 °C with flow rate 800 L/h. Annotations were assigned based on computational interpretation of MS signals. 18:1(d9) sphingomyelin (spiked into samples as Avanti SPLASH prior to extraction) was utilized for semiquantitative assessment of ceramides and hexosylceramides due to the similarity in structure, comparable ionization potential in positive ion mode, and similar retention time. 

### 2.5. Data Analysis and Statistics

For each sample, raw data files were converted to cdf format, and matrix of molecular features defined by retention time and mass (*m*/*z*) was generated using XCMS software [21] in R for feature detection and alignment. The centWave algorithm was used for LC-MS data. Features were grouped using RAMClustR [22]), with normalization set to ‘TIC’ (total ion current). LC-MS data were annotated by searching against an in-house spectra and retention time database using RAMSearch. RAMClustR was used to call the findMain [23] function from the interpretMSSpectrum package to infer the molecular weight of each LC-MS compound and annotate the mass signals. The complete MS spectrum and a truncated MSE spectrum were written to a mat format for import to MSFinder [24]. The MSE spectrum was truncated to only include masses with values less than the inferred M plus its isotopes, and the .mat file precursor ion was set to the M + H ion for the findMain inferred M value. These .mat spectra were analyzed to determine the most probable molecular formula and structure. MSFinder was used to perform a spectral search against the MassBank database. All results were imported into R and a collective annotation was derived with prioritization of RAMSearch > MSFinder mssearch > MSFinder structure > MSFinder formula > findMain M. Annotation confidence was reported as described [25]. All R work was performed using R 3.3.1 [26]. All statistical analysis and figure generation was performed in R. An unpaired Student’s *t*-test with false discovery rate (FDR) correction was performed with FDR-corrected *p*-values < 0.05 considered significant in comparing AfAm- and EuAm-men. 

## 3. Results 

**Figure 2:**
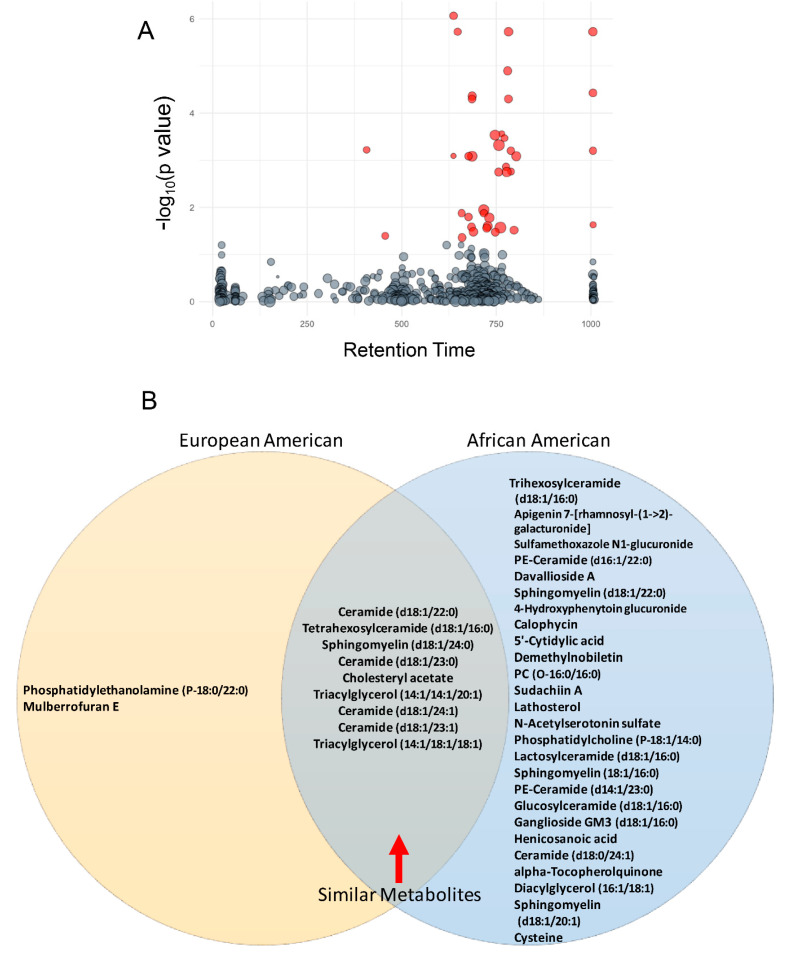
Plasma lipid association with aggressiveness in PCaP. (**A**) Metabolomic analysis revealed 35 plasma lipids were associated significantly with aggressiveness after FDR correction (red bubbles). Y axis displays ANOVA result (-log(*p*-value)). Bubble size is proportional to the log of the spectral signal intensity. (**B**) European-American and African-American men in this cohort shared nine common lipids, which included six sphingolipids that were associated with PCa aggressiveness. (*four chemical formulas were identified for which no chemical compound could be identified).

Discovery metabolomic analyses were performed on plasma samples from 159 men with PCa (Table 1) with similar ages and severity distributions to determine if there was a common set of lipids associated with PCa aggressiveness (see Section 2). Initial analysis compared peak intensities of circulating small molecules/metabolites with tumor aggressiveness [20]. Thirty-five metabolites were associated significantly with aggressiveness (Figure 2A and Table S1) after FDR correction. All but three of these metabolites were molecular species of five distinct lipid classes that included phospholipids, sphingolipids, triglycerides, unesterified fatty acids, and cholesterol/lathosterol. Fifteen of the molecular species most associated with PCa aggressiveness were sphingolipids, including the top five by significance (Table S1). Those with the strongest associations were sphingomyelins and glycosphingolipids, which included tetrahexosylceramide (d18:1/16:0) and trihexosylceramide (d18:1/16:0). Metabolomic/lipidomic data from AfAm- and EuAm-men were analyzed separately to explore if there were racial/ethnic specific metabolites associated with PCa status. An unpaired Student’s *t*-test determined the molecules that were significantly increased between Grade Group 1 (low aggressiveness) versus Grade Groups 2–5 (moderate to high aggressiveness) in both AfAm patients and EuAm patients. The significant metabolites were then compared between the two groups in the Venn Diagram. Thirty-three metabolites were significant in AfAm-men compared to 11 metabolites in EuAm-men (Figure 2B). Of the metabolites observed in both groups, nine were common, and these included previously described metabolites involved in glycosphingolipid synthesis. Of those exclusive to EuAm-men, one was a phospholipid containing saturated fatty acids at the sn-1 and sn-2 positions and one compound (annotated as Mulberrofuran E) typically found in fruits. Twenty-four additional metabolites were associated with PCa severity only in AfAm men, and these metabolites included numerous molecular species in sphingolipid, phospholipid, and cholesterol metabolism. 

Index values of the Variable Importance in Projection (VIP) in partial least squares-discriminant analysis (PLS-DA) were used to evaluate the capacity of individual molecules to distinguish low from intermediate-high aggressiveness. Similar to data shown in Figure 2, PLS-DA of the entire data set identified the same molecular species of sphingomyelins and glycosphingolipids, with five of the top six having the most significant VIP scores (Figure S1). Together, these data suggest that these sphingolipids may serve as a potential marker for PCa aggressiveness across racial/ethnic populations. 

**Figure 3:**
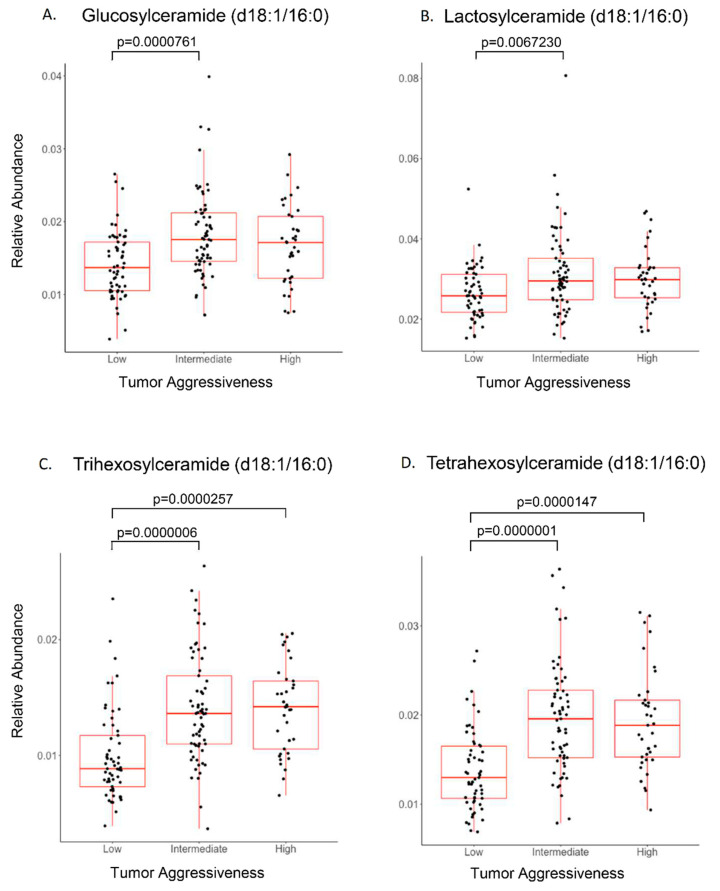
Sphingolipid association with PCa aggressiveness. PCa plasma samples were stratified for low-, intermediate-, and high-aggressive scores (PCaP assigned) and analyzed (ANOVA) for individual sphingolipids. (**A**) Glucosylceramide and (**B**) lactosylceramide were elevated significantly in intermediate-aggressive PCa samples. (**C)** Trihexosylceramide and (**D**) tetrahexosylceramide were elevated significantly in both intermediate- and high-aggressive PCa samples.

Discovery metabolomics were utilized initially as an unbiased approach to determine perturbations in metabolism and molecular networks without an a priori metabolic hypothesis. The data provided by untargeted metabolomics is “compositional data” where individual components (or the signal intensity of individual metabolites) are a proportion of the whole (or total signal). Consequently, these data do not provide a quantitative measure for each metabolite. To partially overcome this limitation, further analyses were conducted using the internal standard mixture of deuterated lipids, which included a deuterated sphingomyelin molecular species (N-oleoyl (d9)-D-erythro-sphingosylphosphorylcholine), to provide semiquantitative measurements of sphingomyelins and glycosphingolipids. Figure 3 illustrates the relative abundance of glucosylceramide, lactosylceramide, and tri- and tetra-hexosylceramides in relation to PCa aggressiveness (20). In addition to glycosphingolipids, ceramides (Figure S2), sphingomyelins, and TAGs (Figure S3) were also elevated in intermediate/high aggressive PCa patients. These data revealed that the relative abundance of specific molecular species of glycosphingolipids were different in low versus intermediate or high aggressive PCa. Only ceramide (d18:1/24:1) exhibited a significant difference between intermediate and high aggressive PCa in the samples analyzed (Figure S2).

**Figure 4:**
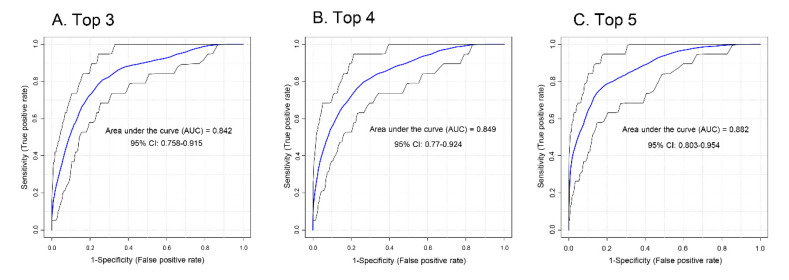
ROC analysis for sphingolipid association with PCa aggressiveness. (**A**) The top three most intense sphingolipids (tetrahexosylceramide (d18:1/16:0), sphingomyelin (d18:0/24:1), and trihexosylceramide (d18:1/16:0)), (**B**) top four (top three plus ceramide (d18:1/24:1)), and (**C**) top five (top four plus ceramide (d18:1/22:0)) sphingolipids were analyzed by ROC for association with aggressiveness.

Receiver operator characteristic curves (ROCs) were generated for the four lipid classes identified (sphingolipids [including glycosphingolipids], saturated phospholipids, cholesterol/lathosterol, and triglycerides) to assess the potential of these lipids to serve as biomarkers for PCa aggressiveness in a binary (low vs. intermediate-high) classifier system (Figure S4A–D). Sphingolipids scored the highest with an area under the curve (AUC) of 0.842 (0.728–0.937, 95% CI) (where 1.000 would indicate no false positives or false negatives). Focusing only on molecular species of sphingolipids, tetrahexosylceramide had AUC 0.815 (0.716–0.896, 95% CI), trihexosylceramide had AUC 0.808 (0.718–0.901, 95% CI), ceramide (d18:1/22:0) had AUC 0.801 (0.722–0.859, 95% CI), and sphingomyelin (d18:0/24:1) had AUC 0.785 (0.694–0.867, 95% CI) (not shown). Combinations of sphingolipids were tested to determine if this improved accuracy. The top three sphingolipids, d18:0/24:1 sphingomyelin, d18:1/16:0 tri and tetrahexosylceramides, yielded AUC 0.842 (CI = 0.758–0.915; Figure 4A). The top four sphingolipids included the addition of d18:1/24:1 ceramide and yielded AUC 0.849 (CI: 0.770–0.924; Figure 4B). Analysis of the top five most significant sphingolipids yielded AUC 0.882 (CI = 0.803–0.954; Figure 4C) after the addition of d18:1/22:0 ceramide. PSA (which is one of three components that give rise to the aggressiveness score) for the same sample set yielded AUC 0.742 (CI = 0.649–0.823, Figure S5). These data suggest that the five-sphingolipid signature may be as accurate or even slightly more as a marker for aggressive PCa cancer than PSA in PCa patients.

**Figure 5:**
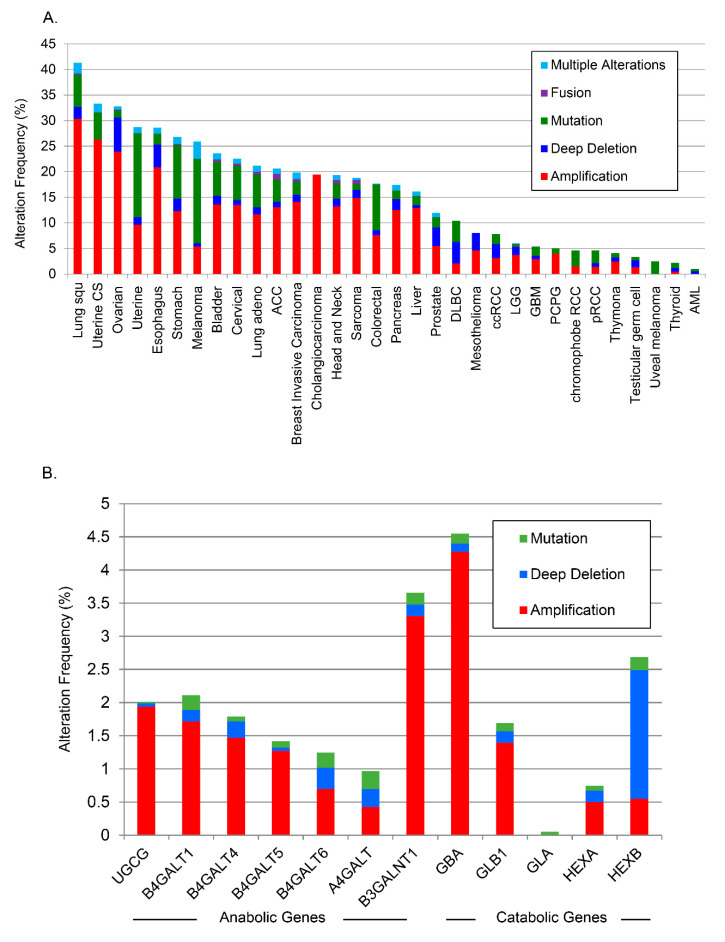

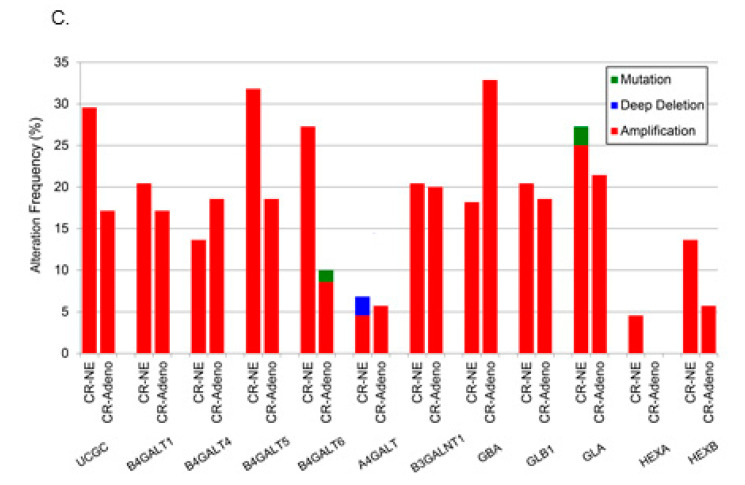
Analysis of changes in glycosphingolipid metabolic genes from TCGA. (**A**) TCGA data were analyzed across cancers for alterations to glycosphingolipid genes, (**B**) by anabolic and catabolic genes in PCa, and (**C**) in castration-resistant PCa.

As the two sphingolipid species most associated with aggressiveness were complex glycosphingolipids (tri- and tetrahexosylceramide), the TCGA PanCancer Atlas online data base was examined using cBioPortal to explore whether there were alterations in the genes involved in the metabolism of these molecules from ceramide. Analyses of the anabolic and catabolic genes in this pathway demonstrated alterations (primarily amplifications) for glycosphingolipid genes across several cancer types, which occurred in ≈12% of PCa cancers (Figure 5A; individual genes analyzed are listed separately in Figure 5B). Lung squamous cell carcinoma had the highest alteration frequency with ≈41% of patients exhibiting alterations in glycosphingolipid pathway genes. High alteration rates (>25%) were found in ovarian, uterine, esophagus, melanoma, and stomach cancers.

A separate analysis of alterations of individual anabolic (Figure 5B) and catabolic genes in glycosphingolipid metabolism in all PCa studies available through cBioPortal [27] demonstrated individual alterations as high as 4.5% throughout the pathway. There was a striking increase in the amplification frequency of *B3GALNT1*, which is responsible for the biosynthesis of tetrahexosylceramides, and a deep deletion profile for *HEXB*, the enzyme that removes sugar moieties from tetrahexosylceramides (Figure 5B). Beltran et al. carried out whole exome sequencing of metastatic biopsies (114 metastatic tumor specimens) of castration-resistant PCa or neuroendocrine PCa [28]. Further analysis of available data from that study revealed amplifications in most glycosphingolipid biosynthetic genes with *B4GALT5*, which codes for the enzyme that catalyzes the synthesis of lactosylceramide, amplified in ≈30% of those castration resistant biopsies (Figure 5C). The combined data from the PCaP and TCGA analyses suggest that putative biomarker patterns described above may correlate with genetic alterations in PCa tissue.

## 4. Discussion 

The emergence of unbiased molecular analytic approaches, such as metabolomics and lipidomics, is providing new opportunities to discover biomarkers that could improve the detection and clinical management of numerous cancers. However, metabolic networks and resulting biomarkers have not been identified that enable early detection, prognosis, or prediction of PCa status, which could enhance disease detection and reduce overdiagnosis and overtreatment of indolent PCa. We used a discovery metabolomic approach, through which we sought to identify metabolic network(s) and biomarkers that that could discriminate PCa aggressiveness in both AfAm- and EuAm-men. Several sphingolipids, specifically glycosphingolipids such as trihexosylceramide and tetrahexosylceramide, proved different between Grade Group 1 and Grade Groups 2–5 in treatment-naïve PCa patients. These findings were further clarified utilizing a deuterated sphingomyelin internal standard and demonstrated differences in circulating levels of four glycosphingolipids when comparing low to intermediate and high aggressive PCa. In contrast, the only lipid to exhibit a significant differences when intermediate and high aggressive groups were compared was ceramide (d18:1/24:1). 

A limitation of these data is there were few men in the aggressive categories, with only 37 of the 159 men belonging to the high aggressive group, which include 25 and 1 in Grade Groups 4 and 5, respectively. This paucity of men with aggressive PCa likely decreased our capacity to differentiate between intermediate and aggressive PCa phenotypes. The concept that this biochemical pathway may also be important in delineating more severe forms of PCa is supported by a recent study that examined the association of plasma lipidomic signatures with clinical outcomes in men with metastatic castration-resistant PCa [29]. Of the 19 lipids associated with overall survival, 12 were sphingolipids including the four glycosphingolipids. That study was able to predict overall survival times in those men using a validated three lipid signature. In our study, both PLS-DA and ROC analyses validated the capacity of similar biomarkers to distinguish clinical difference in treatment naïve clinically localized PCa. A five-sphingolipid signature achieved AUC 0.88, which indicated an 88% chance that the model, if validated, could distinguish between low and intermediate/high aggressiveness PCa. Similar analyses performed with PSA, which is a component of the aggressiveness score, demonstrated an AUC of 0.742. An additional limitation of the current analyses is that these data have not been corrected for comorbidities, dietary patterns, medications, or overall survival as in the study by Lin et al. [29]. These data, taken together with the study by Lin et al. [29], suggest that glycosphingolipid analysis may have the capacity to improve PCa management across the clinical spectrum. A clear next step from this study is to determine accurate concentrations of these molecules under a variety of clinical conditions. 

Our TCGA analysis demonstrated alterations in genes associated with glycosphingolipid metabolism in several different cancers, and specifically in castration-resistant PCa. Admittedly, there were amplifications of both anabolic and catabolic genes. Studies in exosomes from androgen resistant prostate cancer cell lines demonstrated increased levels of sphingolipids and glycosphingolipids, as compared to their parent cells [30]. Lin et al. also demonstrated increases in glycosphingolipids in plasma from castration-resistant PCa patients. Together these studies suggest that glycosphingolipids, and potentially levels of their metabolizing genes, correlate with castration-resistant and aggressive PCa. Future studies are needed to define the source of glycosphingolipids in the plasma of PCa patients.

A key question from these data are why this metabolic network has not been a prominent feature of other metabolomics and lipidomic studies. Two recent systematic reviews of metabolomics biomarkers in PCa identified very few studies where sphingolipids were observed in their analyses [12,13]. Studies by Clos-Garcia et al. demonstrated decreased levels of ceramides (d18:1/16:0, d18:1/20:0, and d18:1/22:0) in urine extracellular vesicles from stage 3 PCa patients as compared to patients with stage 2 disease [31]. Metabolic fingerprinting of urine from PCa patients and healthy volunteers demonstrated increased sphingosine levels in PCa patients [32]. Sphingosine levels were elevated in tissues from PCa patients compared to patients with benign prostatic hyperplasia [33]. Serum metabolites analyzed from PCa patients in studies by Huang et al. demonstrated increases in stearoyl-, euricoyl-, and myristoyl-sphingomyelin in T3 PCa patients [34]. While these studies demonstrate the potential importance for some sphingolipids in PCa, none of the studies identified glycosphingolipids in their metabolic screens.

De novo ceramide generation occurs in the ER and ceramides have been implicated in cellular stress [35], senescence [36], cell cycle arrest [37], aging [38], and in response to chemotherapy [39] (ceramide biology reviewed in [40]. Glycosphingolipids are generated in the Golgi and transported to the plasma membrane. Our analyses suggest the potential for sequestration of this molecular species of ceramide, both molecularly and localization, into more complex glycosphingolipids. Complex glycosphingolipids, specifically glucosylceramide, have been implicated in upregulation of MDR proteins and MDR in numerous types of cancer cells [41,42]. More recent studies have demonstrated that glucosylceramide synthase (GCS) increased levels of globosides (circulating glycosphingolipids) resulting in the expression of *MDR1*, via cSrc and beta-catenin [43]. Genetic and epigenetic alterations in lipid metabolizing genes have been documented in PCa [44]. The analysis of anabolic and catabolic genes in this study suggest that numerous cancers including lung, ovarian, uterine, esophagus, melanoma, stomach, and the most aggressive forms of PCa may be impacted by the glycosphingolipid pathway. The aforementioned mechanistic studies coupled with the current observations provide further evidence that glycosphingolipid biosynthesis plays an important role in several cancers and glycosphingolipids may serve as important plasma biomarkers for cancer aggressiveness. 

Another important observation from this study is that there appears to be a large set of putative biomarkers that are primarily observed in AfAm-men when circulating metabolites are analyzed by race/ethnic group. This group of metabolites contains numerous molecules involved in sphingolipid metabolism, but also metabolites associated with cholesterol and saturated phospholipids, as well as nucleotide and flavonoid metabolism. Together these data demonstrate the potential for novel diagnostic and potentially prognostic biomarker in aggressive PCa. 

## 5. Conclusions

It is clear from the current study that specific glycosphingolipid species appear to be associated with PCa aggressiveness in this cohort of PCaP subjects. Future studies will be geared at testing the diagnostic and prognostic potential for glycosphingolipids in PCa in larger cohorts of patients, with additional to determine the source of these lipids and if there are race/ethnic specific metabolic/lipidomic biomarkers associated with PCa aggressiveness.

## Figures and Tables

**Table 1:** Demographics for the PCaP cohort examined.

Demographics (N = 159)			
Reported Race		African American	European American
		80	79
Age at Diagnosis	Avg. (S.D.)	62 (9)	66 (8)
	≤50	11	2
	51–55	9	8
	56–60	15	9
	61–65	14	19
	66–70	17	15
	71–75	12	15
	>75	2	11
Measures of Severity			
Aggressiveness	Low	27	30
	Intermediate	37	28
	High	16	21
Grade Group	1	29	31
	2	25	25
	3	15	8
	4 + 5	11	15

## Data Availability

The data that support the findings of this study are available from PCaP--NC management team. Restrictions apply to the availability of these data, which were with specific PCaP approval for this study.

## Supplementary Materials

The following are available online at https://www.mdpi.com/2218-273X/10/10/1393/s1, Figure S1: Partial least squares-discriminant analysis (PLS-DA); Figure S2: Sphingolipid association with PCa aggressiveness; Figure S3. Sphingomyelins and triglycerides association with PCa aggressiveness; Figure S4. ROC analysis of PSA vs. aggressiveness; Figure S5. ROC analysis of PSA vs aggressiveness. PSA was analyzed by ROC for association with aggressiveness. Table S1. Plasma metabolites identified that significantly associated with PCA aggressiveness.
