# Supplementary material for: Identification of Plasma Glycosphingolipids as Potential Biomarkers for Prostate Cancer (PCa) Status
Source: Biomolecules. 2020 Sep 30;10(10):1393. doi: 10.3390/biom10101393 (PMC7600119; doi:10.3390/biom10101393)
Supplement: Supplementary file 1 [file biomolecules-10-01393-s001.zip › Figure S2 legend.pdf]

Figure S2. Sphingolipid association with PCa aggressiveness. PCa plasma samples were stratified for low-, intermediate- and high-aggressive scores (PCaP assigned) and analyzed (ANOVA) for individual sphingolipids. Cer: ceramide, CerP: ceramide-1-phosphate, PECer: ceramide phosphatidylethanolamine.
