# Supplementary figures and images for: Identification of Plasma Glycosphingolipids as Potential Biomarkers for Prostate Cancer (PCa) Status
Source: Biomolecules. 2020 Sep 30;10(10):1393. doi: 10.3390/biom10101393 (PMC7600119; doi:10.3390/biom10101393)

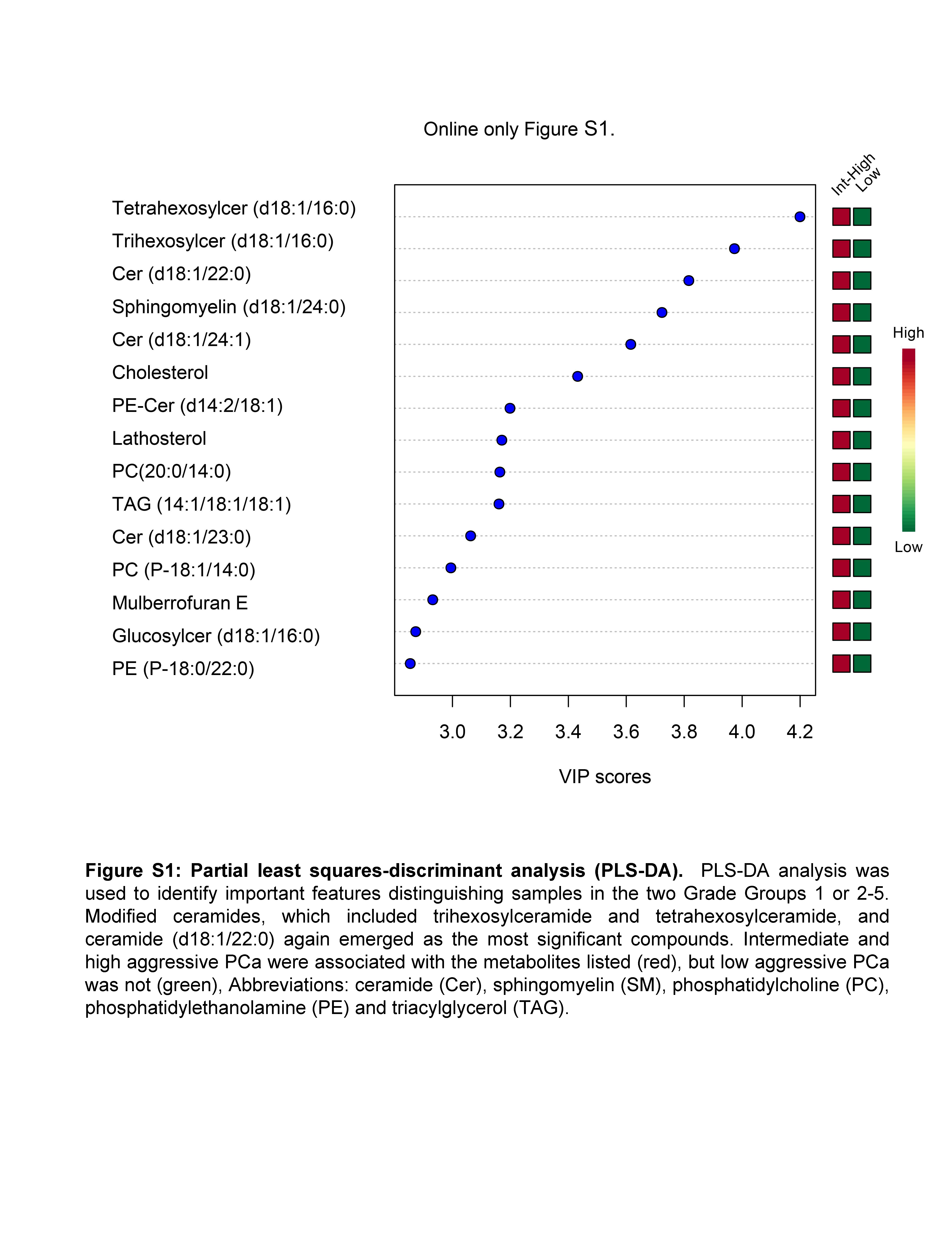

Supplement: Supplementary file 1 [file biomolecules-10-01393-s001.zip › Figure S1.tif]

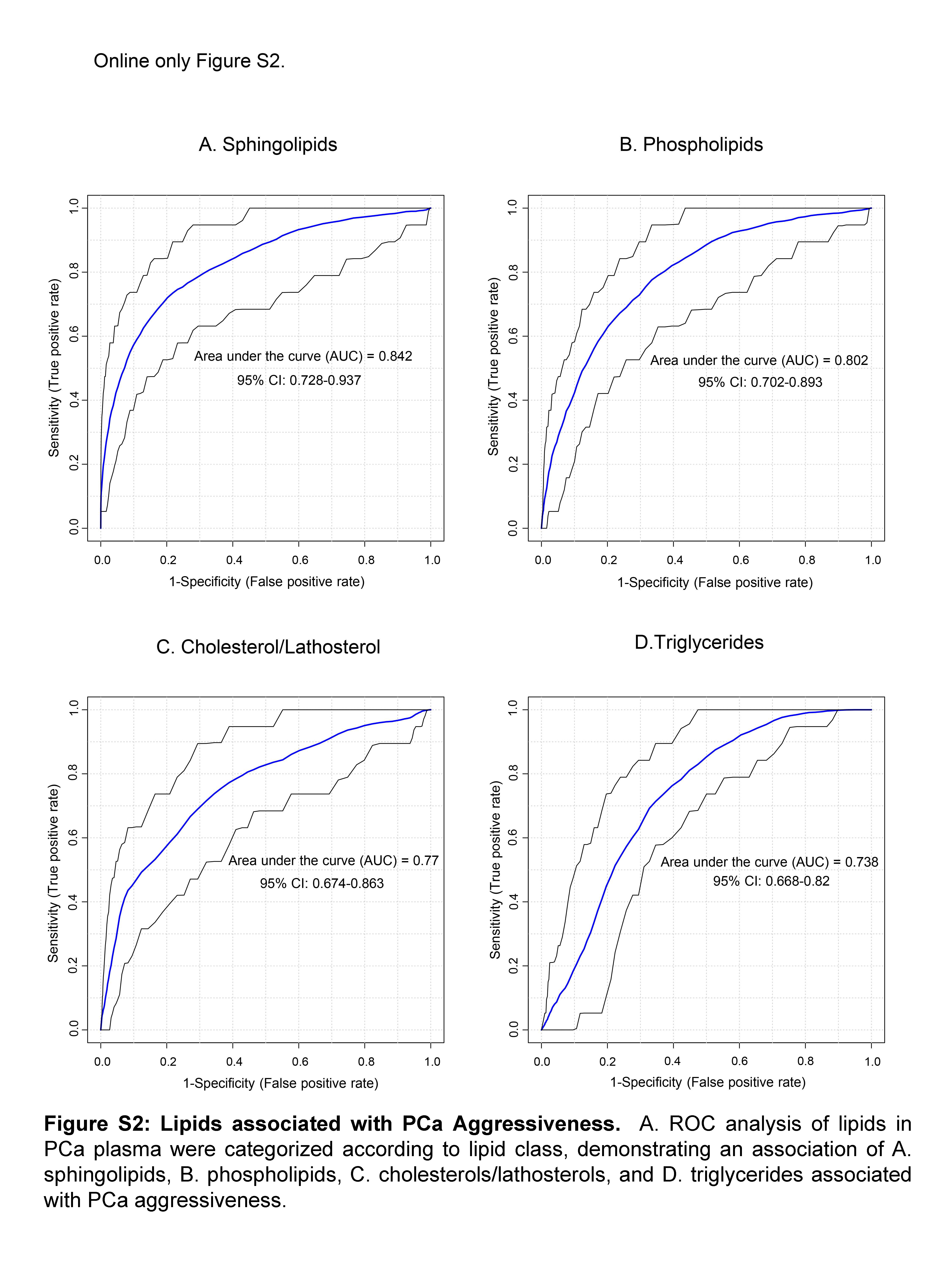

Supplement: Supplementary file 1 [file biomolecules-10-01393-s001.zip › Figure S4.tif]

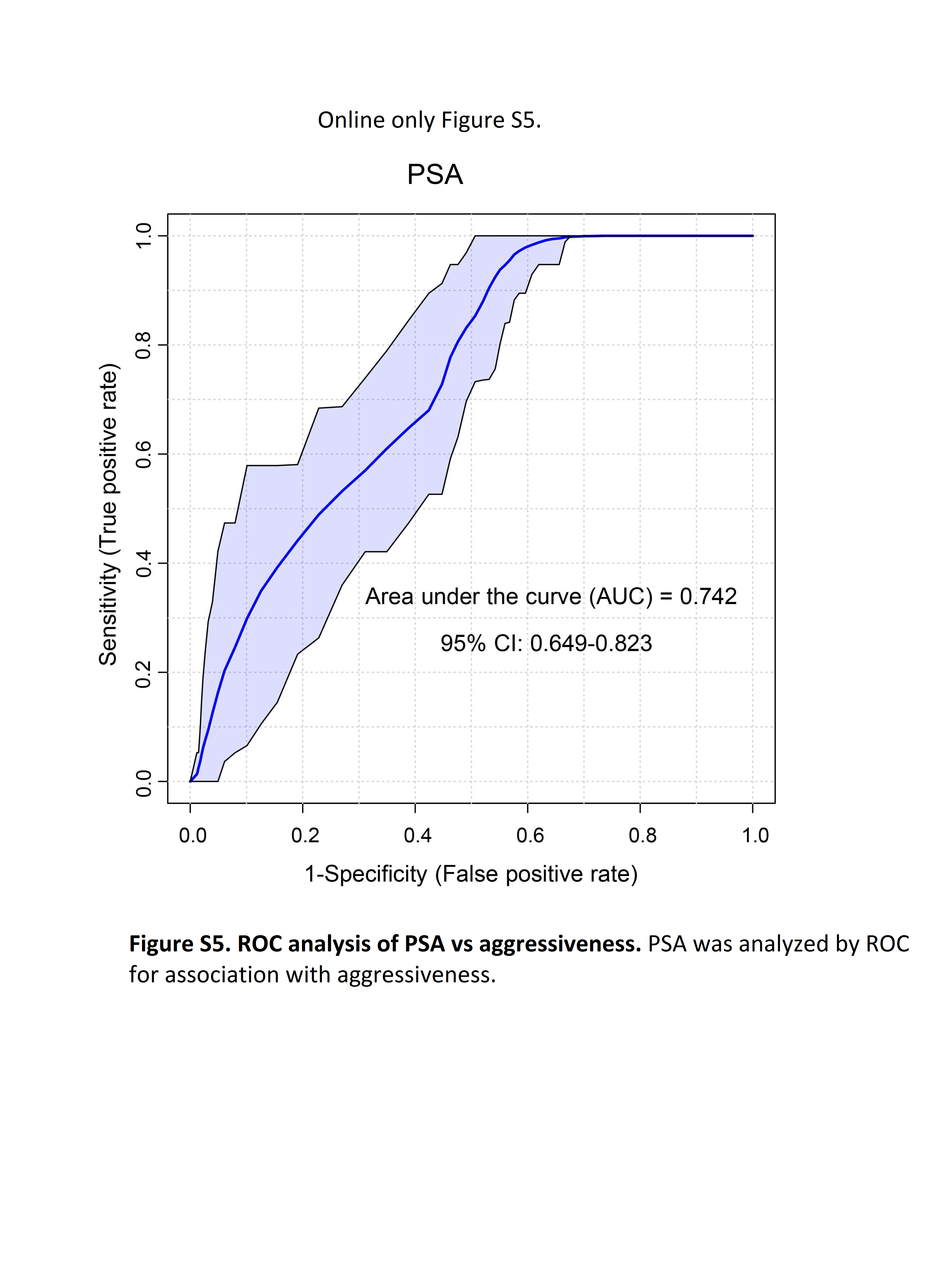

Supplement: Supplementary file 1 [file biomolecules-10-01393-s001.zip › Figure S5.tiff]
